# Comparative plasma and tissue distribution of Sun Pharma’s generic doxorubicin HCl liposome injection versus Caelyx^®^ (doxorubicin HCl liposome injection) in syngeneic fibrosarcoma-bearing BALB/c mice and Sprague–Dawley rats

**DOI:** 10.1007/s00280-017-3278-9

**Published:** 2017-03-27

**Authors:** Vinod Burade, Subhas Bhowmick, Kuntal Maiti, Rishit Zalawadia, Deepak Jain, Thennati Rajamannar

**Affiliations:** 1 Sun Pharma Advanced Research Company Ltd., 17 B Mahal Industrial Estate, Mahakali Caves Road, Andheri (East), Mumbai, Maharashtra 400 093 India; 2 Sun Pharmaceutical Industries Ltd., Sun Pharma Advanced Research Centre (SPARC), Tandalja, Vadodara, Gujarat 390 020 India

**Keywords:** Anthracycline, Doxorubicin HCl liposome injection, Pharmacokinetics, Plasma distribution, Preclinical, Tissue distribution

## Abstract

**Purpose:**

The liposomal formulation of doxorubicin [doxorubicin (DXR) hydrochloride (HCl) liposome injection, Caelyx^®^] alters the tissue distribution of DXR as compared with nonliposomal DXR, resulting in an improved benefit-risk profile. We conducted studies in murine models to compare the plasma and tissue distribution of a proposed generic DXR HCl liposome injection developed by Sun Pharmaceuticals Industries Limited (SPIL DXR HCl liposome injection) with Caelyx^®^.

**Methods:**

The plasma and tissue distributions of the SPIL and reference DXR HCl liposome injections were compared in syngeneic fibrosarcoma-bearing BALB/c mice and Sprague–Dawley rats. Different batches and different lots of the same batch of the reference product were also compared with each other.

**Results:**

The SPIL and reference DXR HCl liposome injections exhibited generally comparable plasma and tissue distribution profiles in both models. While minor differences were observed between the two products in some tissues, different batches and lots of the reference product also showed some differences in the distribution of various analytes in some tissues. The ratios of estimated free to encapsulated DXR for plasma and tissue were generally comparable between the SPIL and reference DXR HCl liposome injections in both models, indicating similar extents of absorption into the tissues and similar rates of drug release from liposomes.

**Conclusions:**

The plasma and tissue distribution profiles of the SPIL and reference DXR HCl liposome injections were shown to be generally comparable. Inconsistencies between the products observed in some tissues were thought to be due to biological variation.

## Introduction

The use of doxorubicin (DXR), a potent chemotherapeutic agent, is limited in the clinical setting by its toxicity [1]. The cardiotoxicity caused by DXR is of special concern. Doxorubicin hydrochloride (HCl) liposome injection is a liposomal formulation of DXR that alters the plasma and tissue distribution of DXR, leading to an at least comparable efficacy and an improved toxicological profile over nonliposomal DXR [2–4].

Doxorubicin HCl liposome injection received approval from the US Food and Drug Administration (FDA) in 1995 for the treatment of AIDS-related Kaposi’s sarcoma. Since then, it has received worldwide approval for the treatment of multiple myeloma (combination therapy with bortezomib) and ovarian carcinoma. In the European Union, it is additionally approved for patients with breast cancer who are at increased risk of DXR-associated cardiotoxicity [2, 5]. Janssen is currently marketing DXR HCl liposome injection as Doxil^®^ in the US and Japan and as Caelyx^®^ elsewhere.

Sun Pharmaceutical Industries Limited (SPIL) has developed a generic DXR HCl liposome injection (SPIL DXR HCl liposome injection). In February 2012, the FDA temporarily allowed the importation of Sun Pharma’s domestic liposomal DXR to cope with a Doxil^®^ drug shortage, and because there were no approved generic alternatives [6]. One year later, SPIL DXR HCl liposome injection was formally approved by the FDA.

This study was one of a program of studies, conducted in line with European Medicines Agency (EMA) guidance, to demonstrate similarity between the SPIL DXR HCl liposome injection and Caelyx^®^. The program included physicochemical equivalence studies (structure, content and stability of liposomes in vitro and in vivo), which confirmed that the two liposomal forms are similar (SPIL data on file).

Caelyx^®^ alters the plasma and tissue distribution of DXR, resulting in an improved benefit-risk profile as compared with nonliposomal DXR. Therefore, SPIL DXR HCl liposome injection must achieve comparable plasma and tissue distribution in humans to be considered truly comparable to Caelyx^®^ [2, 7].

Previously, we demonstrated that the preclinical antitumour efficacy and toxicity profile and the extent of total DXR absorption of the SPIL DXR HCl liposome injection were comparable to Caelyx^®^ in relevant mouse models of cancer (Burade et al. Paper accepted by *BMC Cancer* subject to revision).

The objectives of the studies presented in this paper were to compare the plasma and tissue distribution of the SPIL DXR HCl liposome injection with the reference product (Caelyx^®^) following single intravenous injection in syngeneic fibrosarcoma-bearing BALB/c mice and Sprague–Dawley rats. The plasma and tissue distribution of different batches and different lots of the same batch of the reference product were also compared with each other in Sprague–Dawley rats.

## Materials and methods

### Drug administration

The SPIL DXR HCl liposome injection (SPIL, Halol, India) and reference DXR HCl liposome injection (Caelyx^®^, Janssen-Cilag International NV, Beerse, Belgium) were stored at 2–8 °C. The SPIL and reference DXR HCl liposome injections contained 2 mg/mL of the active ingredient, DXR HCl. The SPIL and reference DXR HCl liposome injections also contained *N*-(carbonyl-methoxypolyethylene glycol 2000)-1,2-distearoyl-sn-glycerol-3-phosphoethanolamine (mPEG-DSPE), hydrogenated soy phosphatidylcholine (HSPC), cholesterol, ammonium sulphate, l-histidine as a buffer, hydrochloric acid and/or sodium hydroxide for pH control, sucrose to maintain isotonicity and water for injection. All products were either used at 2 mg/mL, or diluted to the desired concentration in sterile 5% glucose solution.

### Animals

The project proposal for the study was approved by the Institutional Animal Ethics Committee (IAEC), and their recommendations regarding animal care and handling were followed. Male BALB/c mice and male Sprague–Dawley rats were supplied by Laboratory Animal Resources (LAR), Sun Pharma Advanced Research Company Limited (SPARC Ltd.). The BALB/c mice were 6–10 weeks of age at the time of receipt and weighed 25 ± 5 g. The Sprague–Dawley rats were 5–8 weeks of age and weighed 150–180 g at receipt. For BALB/c mice, a veterinary health check was performed before tumour propagation to select healthy animals. For Sprague–Dawley rats, a veterinary health check was performed on day 0 of the study. The BALB/c mice and Sprague–Dawley rats used in the studies were housed in individually ventilated polysulfone cages. The BALB/c mice were housed individually, while Sprague–Dawley rats were housed two to three per cage. Cages were maintained under constant temperature (18–26 °C), humidity (30–70%) and lighting conditions (12 h light and 12 h dark). Animals received reverse osmosis (RO) water supplied by LAR, and Harlan Teklad Rodent Diet 2018 ad libitum.

### Cell lines

WEHI 164 cells suspended in phosphate buffer saline (1–2 × 10^7^ viable cells per mL) were used for the induction of solid tumours. Briefly, male BALB/c mice were shaved with the help of a shaver and hair-removing cream, and inoculated intradermally on the shaved portion of dorsal skin with tumour cells. Calibrated digital Vernier calipers were used to measure tumour diameter in three perpendicular planes, after the tumours became palpable. Tumour volume was calculated using the formula for an ellipse, *V* = *π*/6 (*D*1 × *D*2 × *D*3) mm^3^, where *D*1/*D*2/*D*3 is the diameter (mm) in each of the three different planes.

### Study design

The respective designs of the plasma and tissue distribution studies are shown in Table 1. Fibrosarcoma-bearing male BALB/c mice were screened and selected for randomisation into study groups on the basis of body weight and tumour volume. Selected animals weighed 22.2–30.5 g and had a tumour volume 150 ± 50 mm^3^ at randomisation. Male Sprague–Dawley rats were screened and selected for randomisation into study groups on the basis of body weight, and weighed 190.1–258.0 g at randomisation. The doses of SPIL and reference DXR HCl liposome injections used in these studies (BALB/c mice, 2.4 or 6 mg/kg; Sprague–Dawley rats, 4 or 10 mg/kg) were calculated to be equivalent to 20 or 50 mg/m^2^, respectively, in humans. Intravenous injections were administered on day 0. Blood samples for plasma distribution analysis were collected at 1, 4, 24, 48, 96, 168, 240, 336, or 672 h postinjection (Table 1). Animals were euthanised at this time in order to harvest the heart, skin, liver, kidney, spleen, lung, bone marrow and tumour (BALB/c mice only) for tissue distribution analysis. Plasma was separated from blood samples by centrifugation. For BALB/c mice, plasma samples were pooled from four animals serially, providing three pooled samples for each time point. The tumour (BALB/c mice only), heart, skin, liver, kidney, spleen and lung were dissected out from all animals. Both hind legs were dissected out for bone marrow preparation, which was scrapped and collected after cutting bones longitudinally using scalpel blades. All tissues were blotted on Whatman filter paper No. 1 to remove the blood. For BALB/c mice, samples of each tissue from four animals were pooled, providing three pooled samples per tissue for each time point (except bone marrow, where samples from all 12 animals were pooled for each time point). Tissue homogenates were prepared using a homogenizer. A 20% weight/volume (w/v) tissue homogenate was prepared for tumour (BALB/c mice only), heart, liver, kidney, spleen, lung and bone marrow. For skin, either a 10 or 20% w/v tissue homogenate was prepared (2.4 mg/kg mouse study: a 10% w/v skin homogenate was prepared for all time points except at 96 h; 6 mg/kg mouse study: a 20% w/v skin tissue homogenate was prepared; 4 mg/kg rat study: a 10% w/v skin homogenate was prepared for all time points except at 1 and 4 h; 10 mg/kg rat study: a 20% w/v skin tissue homogenate was prepared). Heart and skin tissues were minced before homogenization.

**Table 1:** Study design

Group no.	Dose groups	Dose (mg/kg)	Concentration (mg/mL)	Dose volume (mL/kg)	Time point (h)	No. of animals per time point
(a) Study groups for the syngeneic fibrosarcoma-bearing BALB/c mouse study (2.4 mg/kg dose)						
1	SPIL DXR HCl liposome injection	2.4	0.6	4	1	12
					4	12
					24	12
					48	12
					96	12
					168	12
					240	12
					336	12
					672	12
2	Reference DXR HCl liposome injection	2.4	0.6	4	1	12
					4	12
					24	12
					48	12
					96	12
					168	12
					240	12
					336	12
					672	12
Total number						216
(b) Study groups for the syngeneic fibrosarcoma-bearing BALB/c mouse study (6 mg/kg dose)						
1	SPIL DXR HCl liposome injection	6	2	3	1	12
					4	12
					24	12
					48	12
					96	12
					168	12
					240	12
					336	12
					672	12
2	Reference DXR HCl liposome injection	6	2	3	1	12
					4	12
					24	12
					48	12
					96	12
					168	12
					240	12
					336	12
					672	12
Total number						216
(c) Study groups for the Sprague–Dawley rat study (4 mg/kg dose)						
1	SPIL DXR HCl liposome injection	4	2	2	1	10
					4	10
					24	10
					48	10
					96	10
					168	10
					240	10
					336	10
					672	10
					24^a^	10
					48^a^	10
					96^a^	10
2	Reference DXR HCl liposome injection (batch 1)	4	2	2	1	10
					4	10
					24	10
					48	10
					96	10
					168	10
					240	10
					336	10
					672	10
					24^a^	10
					48^a^	10
					96^a^	10
3	Reference DXR HCl liposome injection (batch 2)	4	2	2	1	10
					4	10
					24	10
					48	10
					96	10
					168	10
					240	10
					336	10
					672	10
					24^a^	10
					48^a^	10
					96^a^	10
Total number						360
(d) Study groups for the Sprague–Dawley rat study (10 mg/kg dose)						
1	SPIL DXR HCl liposome injection	10	2	5	1	10
					4	10
					24	10
					48	10
					96	10
					168	10
					240	10
					336	10
					672	10
2	Reference DXR HCl liposome injection (lot 1)	10	2	5	1	10
					4	10
					24	10
					48	10
					96	10
					168	10
					240	10
					336	10
					672	10
3	Reference DXR HCl liposome injection (lot 2)	10	2	5	1	10
					4	10
					24	10
					48	10
					96	10
					168	10
					240	10
					336	10
					672	10
Total number						270

*DXR* doxorubicin, *HCl* hydrochloride, *SPIL* Sun Pharmaceutical Industries Limited

^a^Repeat time points

### Experimental outcomes

Plasma drug levels were measured from the plasma samples for each time point by liquid chromatography–tandem mass spectrometry (LC-MS/MS), and reported as micrograms per millilitre (mcg/mL). Tissue drug levels were measured from tissue samples for each time point by LC-MS/MS, and reported as micrograms of DXR per gram of tissue (mcg/g of tissue). Plasma and tissue data were used to calculate the mean peak concentration (*C*_max_) and mean area under the curve from zero (0) hours to time (*t*) (AUC_0–*t*_) of total DXR, encapsulated DXR, free DXR, and doxorubicinol. The ratio of estimated free to encapsulated DXR was defined as the estimated mean *C*_max_ (or AUC_0–*t*_) of free DXR divided by the mean *C*_max_ (or AUC_0–*t*_) of encapsulated DXR.

### Statistical methods

The mean AUCs and 95% confidence intervals of the difference between every two groups were calculated by the Bailer–Satterthwaite method. A difference in AUCs was considered to be statistically significant when abs(*t*_obs_) ≥ *t*_crit_. For mean *C*_max_, the 95% confidence intervals of the differences between every two groups were calculated by unpaired *t*-test. A difference in *C*_max_ was considered to be statistically significant when *p* ≤ 0.05. Zero-hour concentration values were reported (as zero) for the purpose of calculating AUC only. A statistical analysis of bone marrow derived from syngeneic fibrosarcoma-bearing BALB/c mice was not performed, as only 1 value was available for each group (sample was pooled from 12 animals for concentration analysis owing to limited quantity).

## Results

### Plasma distribution of DXR in syngeneic fibrosarcoma-bearing mice

**Fig. 1:**
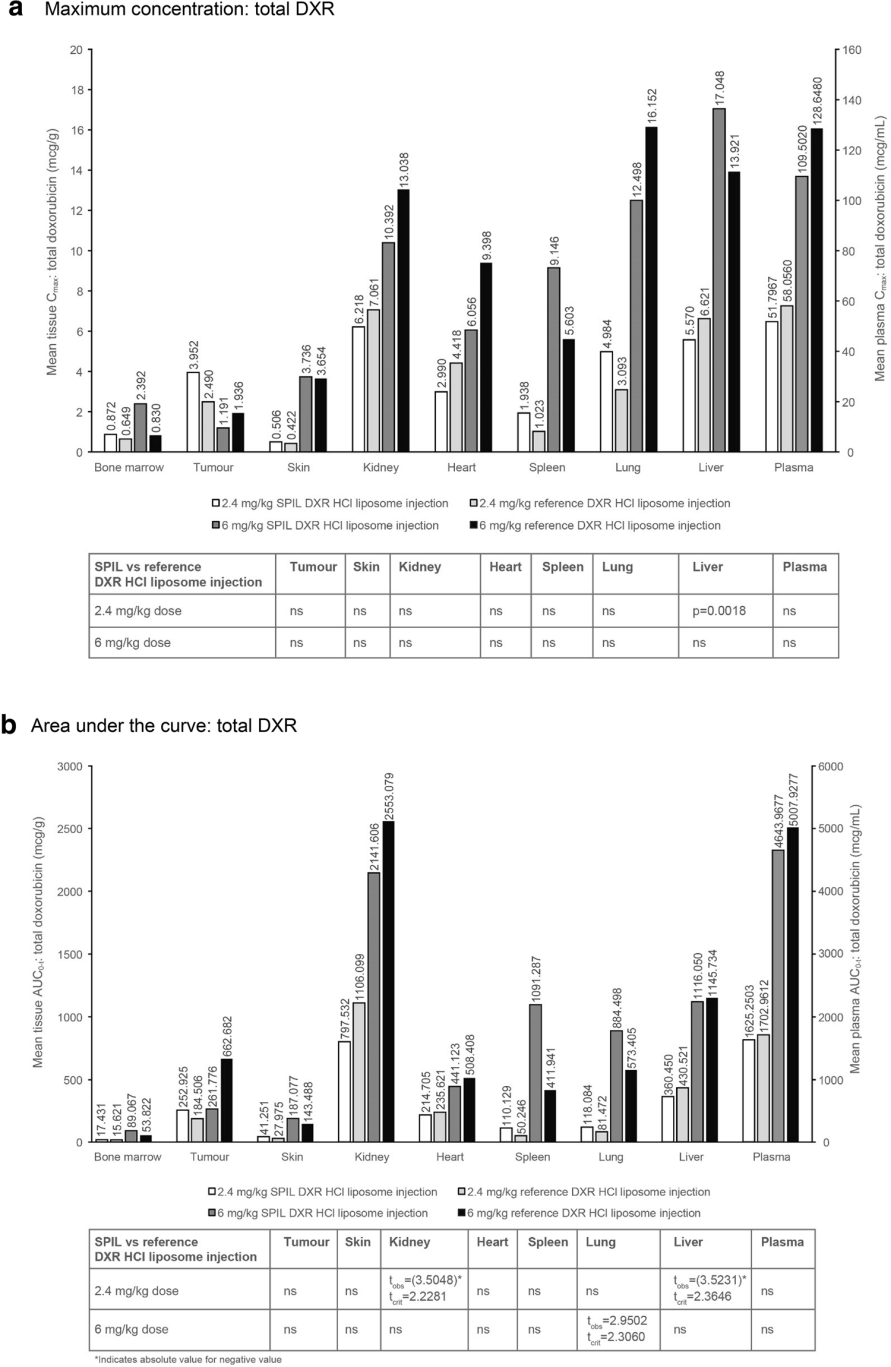

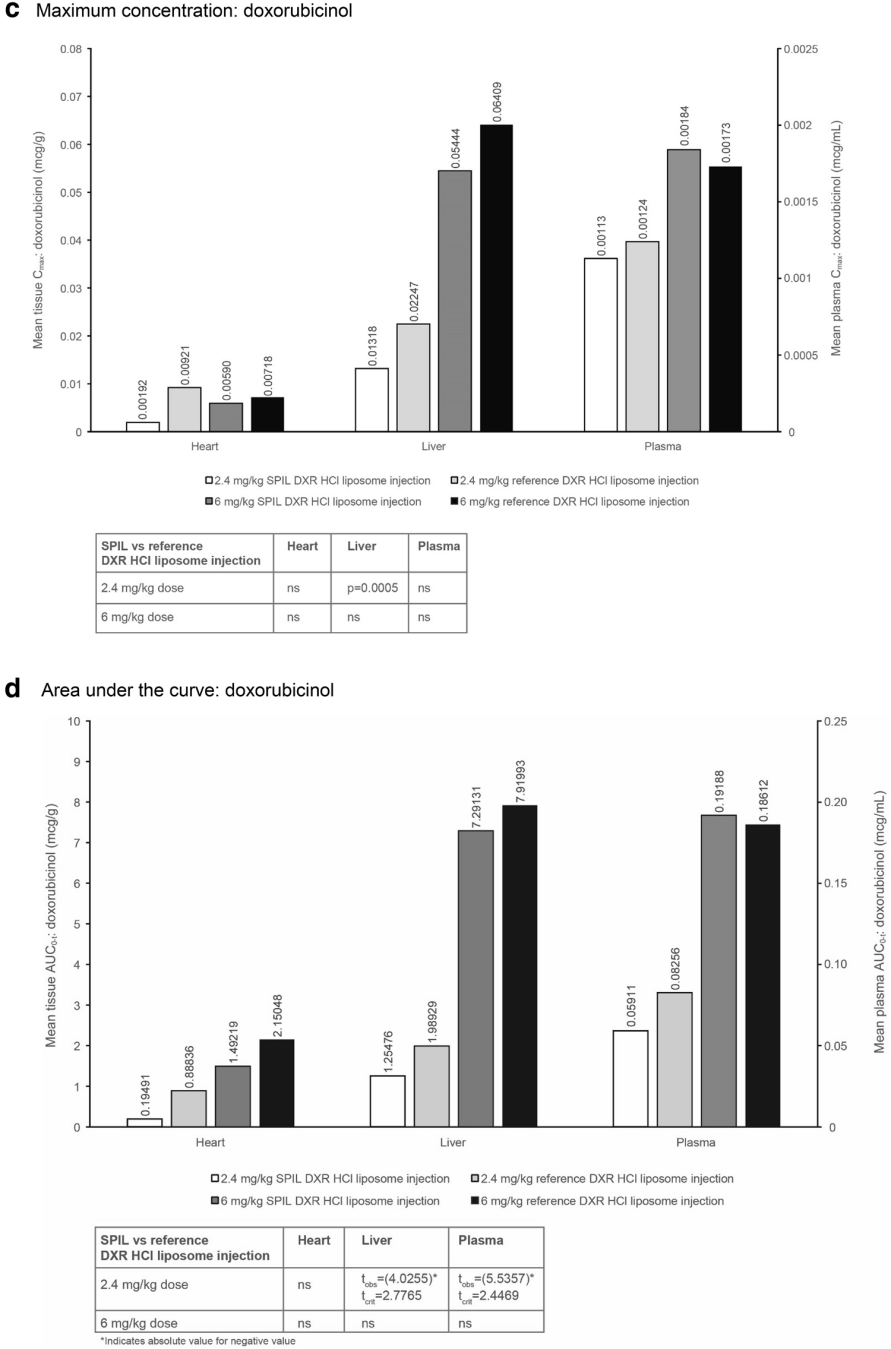
Plasma and tissue distribution of total DXR and doxorubicinol in syngeneic fibrosarcoma-bearing BALB/c mice. **a** *C*_max_ and **b** AUC_0–*t*_ of total DXR after dosing with the SPIL or reference DXR HCl liposome injection. **c** *C*_max_ and **d** AUC_0–*t*_ of doxorubicinol after dosing with the SPIL or reference DXR HCl liposome injection. Twelve animals were analysed per time point. Each plasma sample was pooled from four animals, providing three pooled samples for each time point. Each tissue sample was pooled from four animals, providing three pooled samples per tissue for each time point (except bone marrow, where samples from all 12 animals were pooled for each time point). Differences in *C*_max_ were analysed by unpaired *t*-test. *P* values ≤0.05 were considered significant. Differences in AUC_0–*t*_ were analysed by the Bailer–Satterthwaite method, and a difference was considered to be statistically significant when abs(*t*_obs_) ≥ *t*_crit_. *AUC* area under the concentration–time curve, *C*_*max*_ mean peak concentration, *DXR* doxorubicin, *ns* not significant, *SPIL* Sun Pharmaceutical Industries Limited

Mean plasma *C*_max_ and AUC_0–*t*_ values for total DXR and doxorubicinol, following a single intravenous injection with either the SPIL or reference DXR HCl liposome injection in syngeneic fibrosarcoma-bearing BALB/c mice, are shown in Fig. 1.

#### Maximum concentration

In plasma, the mean *C*_max_ values of total DXR (Fig. 1a), free and encapsulated DXR (data not shown) and doxorubicinol (Fig. 1c) were comparable after 2.4 mg/kg SPIL compared with 2.4 mg/kg reference DXR HCl liposome injection. The mean plasma *C*_max_ values of total DXR (Fig. 1a), free DXR (data not shown) and doxorubicinol (Fig. 1c) were also comparable after 6 mg/kg SPIL and reference DXR HCl liposome injections. However, the mean plasma *C*_max_ of encapsulated DXR was significantly lower after 6 mg/kg SPIL DXR HCl liposome injection compared with the reference DXR HCl liposome injection (*p* = 0.0351; data not shown).

#### Area under the curve

The mean plasma AUC_0–*t*_ values of total DXR (Fig. 1b) and free and encapsulated DXR (data not shown) after 2.4 and 6 mg/kg were comparable between the SPIL and reference DXR HCl liposome injections. However, the mean plasma AUC_0–*t*_ of doxorubicinol was significantly lower after 2.4 mg/kg SPIL DXR HCl liposome injection compared with the reference DXR HCl liposome injection (*t*_crit_ = 2.4469 and *t*_obs_ = −5.5357; Fig. 1d). This was not the case after 6 mg/kg, where the mean plasma AUC_0–*t*_ of doxorubicinol (Fig. 1d) was comparable between the SPIL and reference DXR HCl liposome injections.

### Tissue distribution of DXR in syngeneic fibrosarcoma-bearing mice

Mean tissue *C*_max_ and AUC_0–*t*_ values of total DXR and doxorubicinol (liver and heart) for syngeneic fibrosarcoma-bearing BALB/c mice, following a single intravenous injection with either the SPIL or reference DXR HCl liposome injection, are also shown in Fig. 1.

#### Maximum concentration: total DXR

The mean *C*_max_ of total DXR was comparable between the SPIL and reference DXR HCl liposome injections for all tissues after 2.4 mg/kg, except in the liver, where the *C*_max_ was significantly lower for the SPIL compared with the reference DXR HCl liposome injection (*p* = 0.0018, Fig. 1a). The mean *C*_max_ of total DXR was comparable between the SPIL and reference DXR HCl liposome injections for all tissues after 6 mg/kg.

#### Area under the curve: total DXR

The mean AUC_0–*t*_ of total DXR was not significantly different for most tissues between the SPIL and reference DXR HCl liposome injections after 2.4 or 6 mg/kg (Fig. 1b). The exceptions were the kidney and liver AUC_0–*t*_ values after 2.4 mg/kg, which were significantly lower for the SPIL than for the reference DXR HCl liposome injection (*t*_crit_ = 2.2281 and *t*_obs_ = −3.5048 for the kidney, *t*_crit_ = 2.3646 and *t*_obs_ = −3.5231 for the liver), and the lung AUC_0–*t*_ values after 6 mg/kg, which were significantly higher for the SPIL than for the reference DXR HCl liposome injection (*t*_crit_ = 2.3060 and *t*_obs_ = 2.9502).

#### Maximum concentration: doxorubicinol

The mean *C*_max_ of doxorubicinol was comparable between the SPIL and reference DXR HCl liposome injections in heart tissue, after 2.4 mg/kg. However, the mean liver *C*_max_ was significantly lower for the SPIL compared with the reference DXR HCl liposome injection (*p* = 0.0005; Fig. 1c). The mean *C*_max_ of doxorubicinol was comparable between the SPIL and reference DXR HCl liposome injections in heart and liver tissue, after 6 mg/kg.

#### Area under the curve: doxorubicinol

The mean heart AUC_0–*t*_ values were comparable for the SPIL and reference DXR HCl liposome injections after either 2.4 or 6 mg/kg (Fig. 1d). The mean liver AUC_0–*t*_ values were comparable for 6 mg/kg SPIL and reference DXR HCl liposome injections, but a significantly lower liver AUC_0–*t*_ value was observed after 2.4 mg/kg SPIL compared with 2.4 mg/kg reference DXR HCl liposome injection (*t*_crit_ = 2.7765 and *t*_obs_ = −4.0255). This reflects the same trend observed in plasma.

### Plasma distribution of DXR in Sprague–Dawley rats

**Fig. 2:**
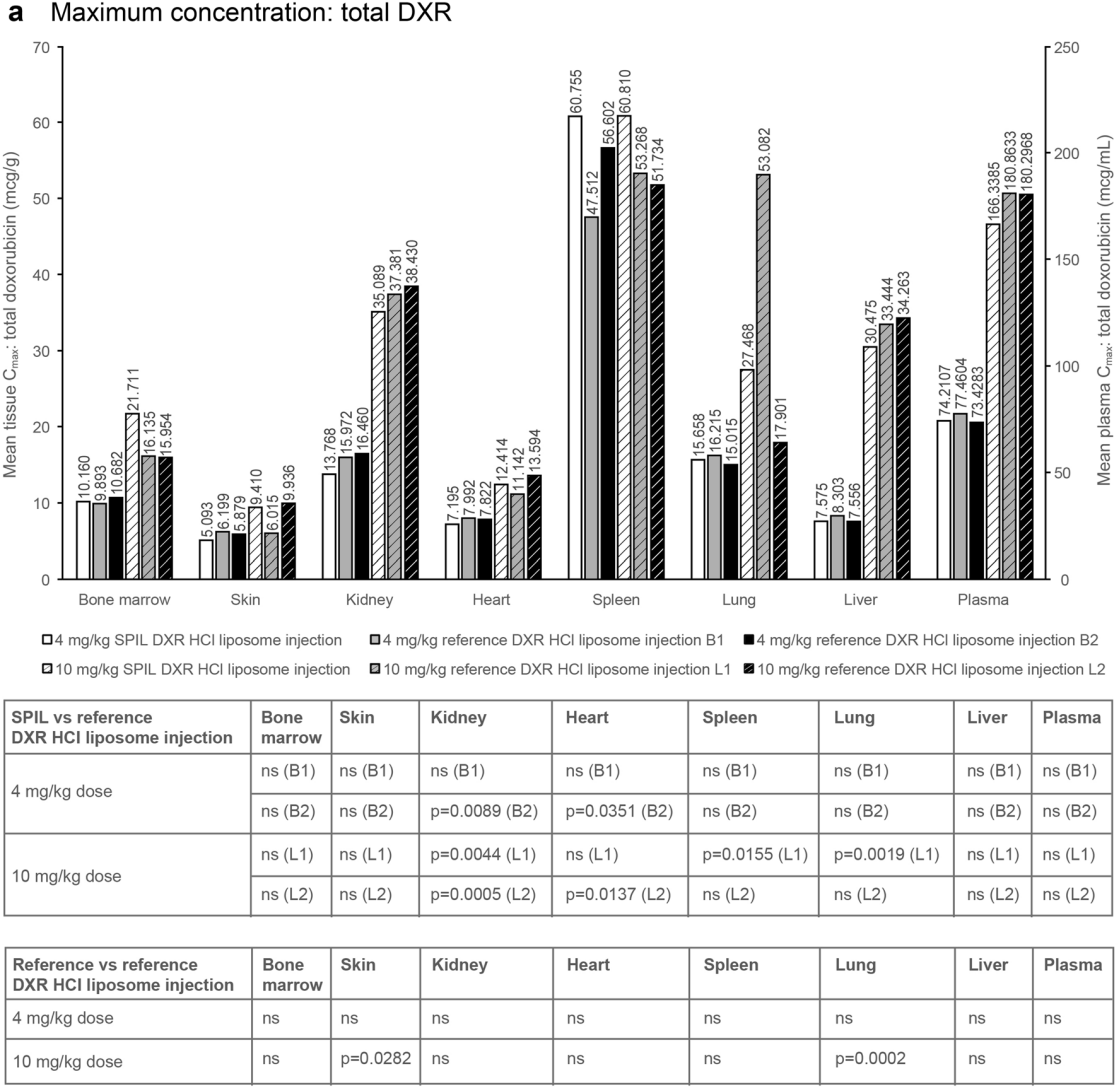

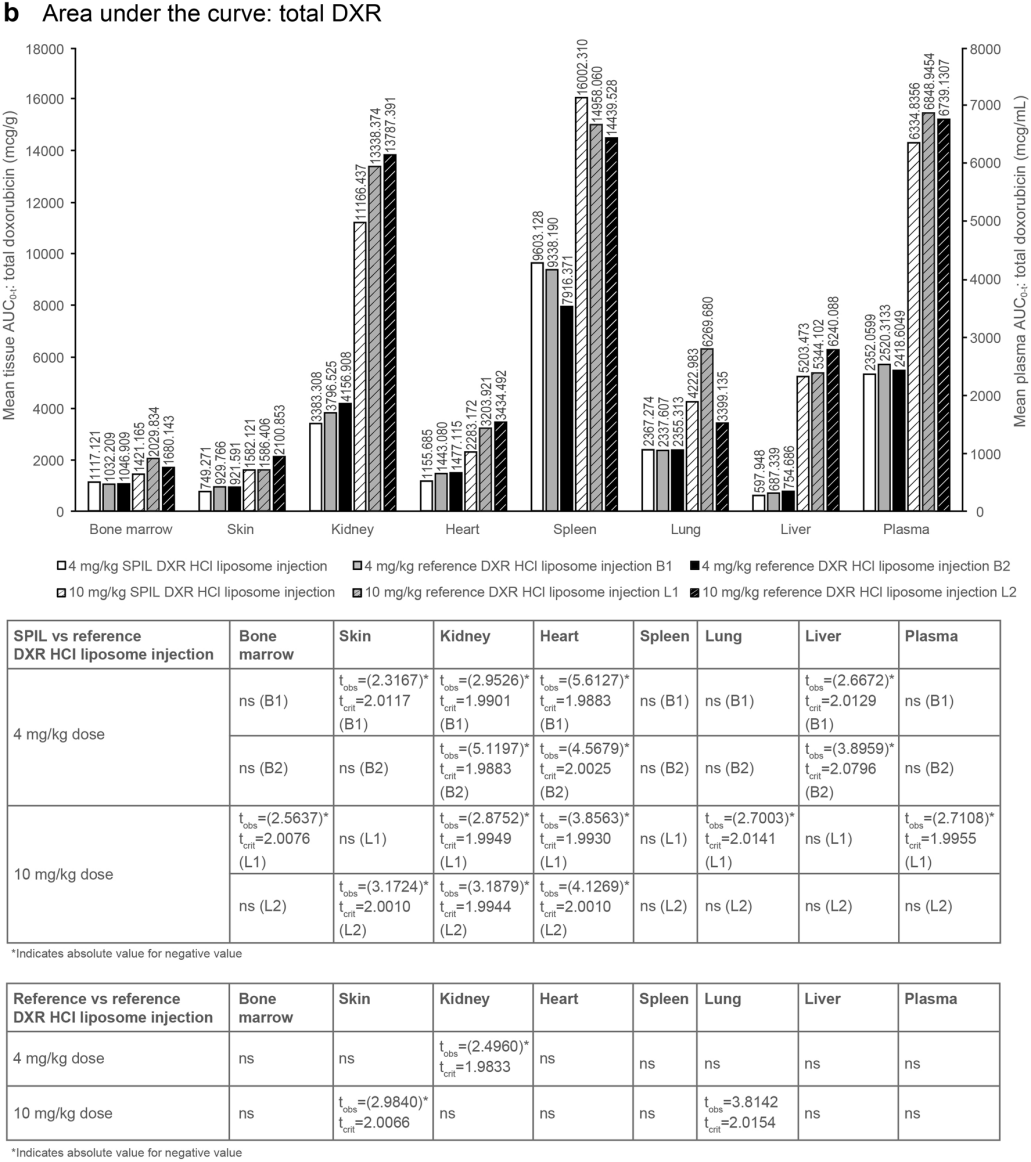

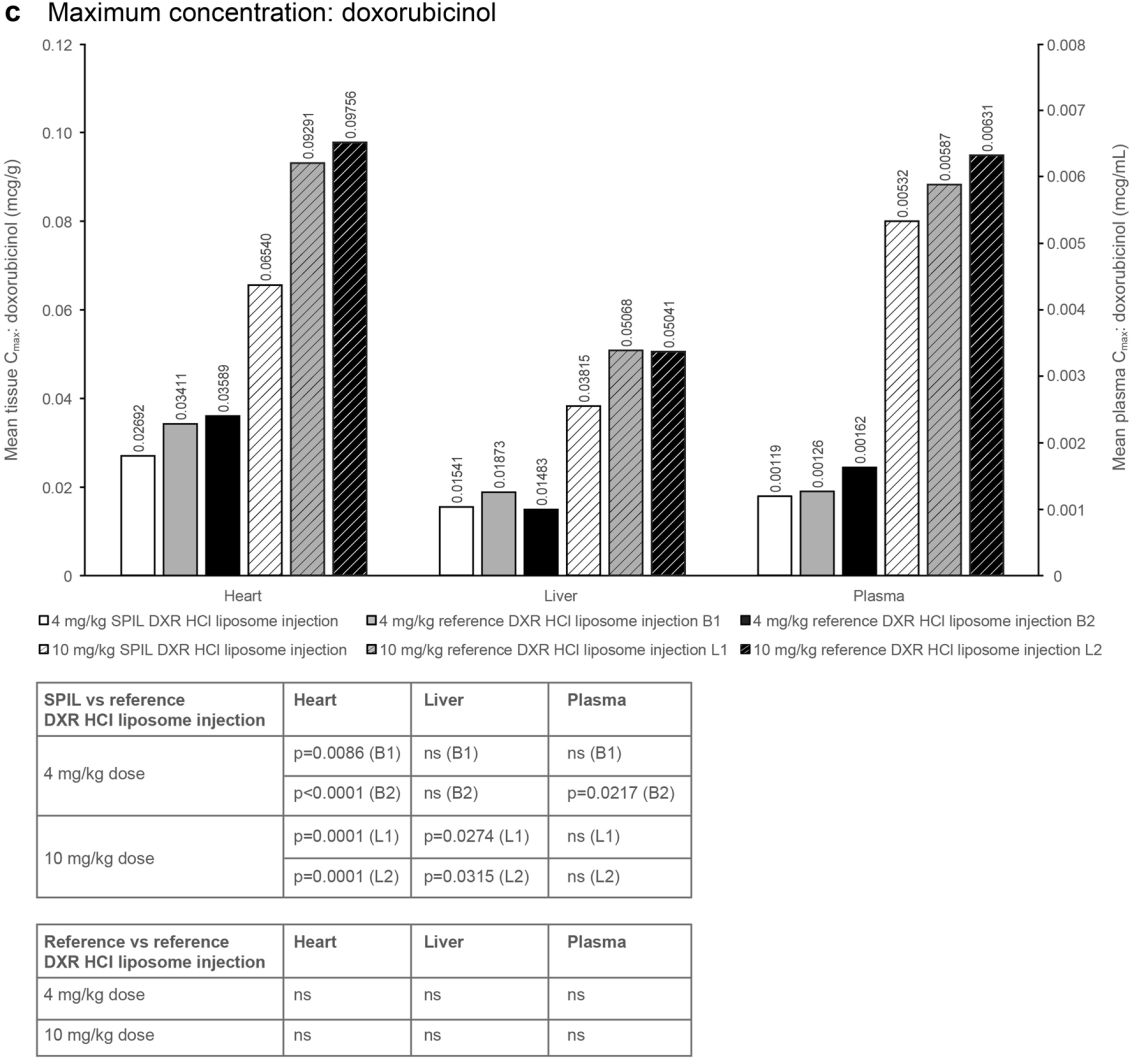

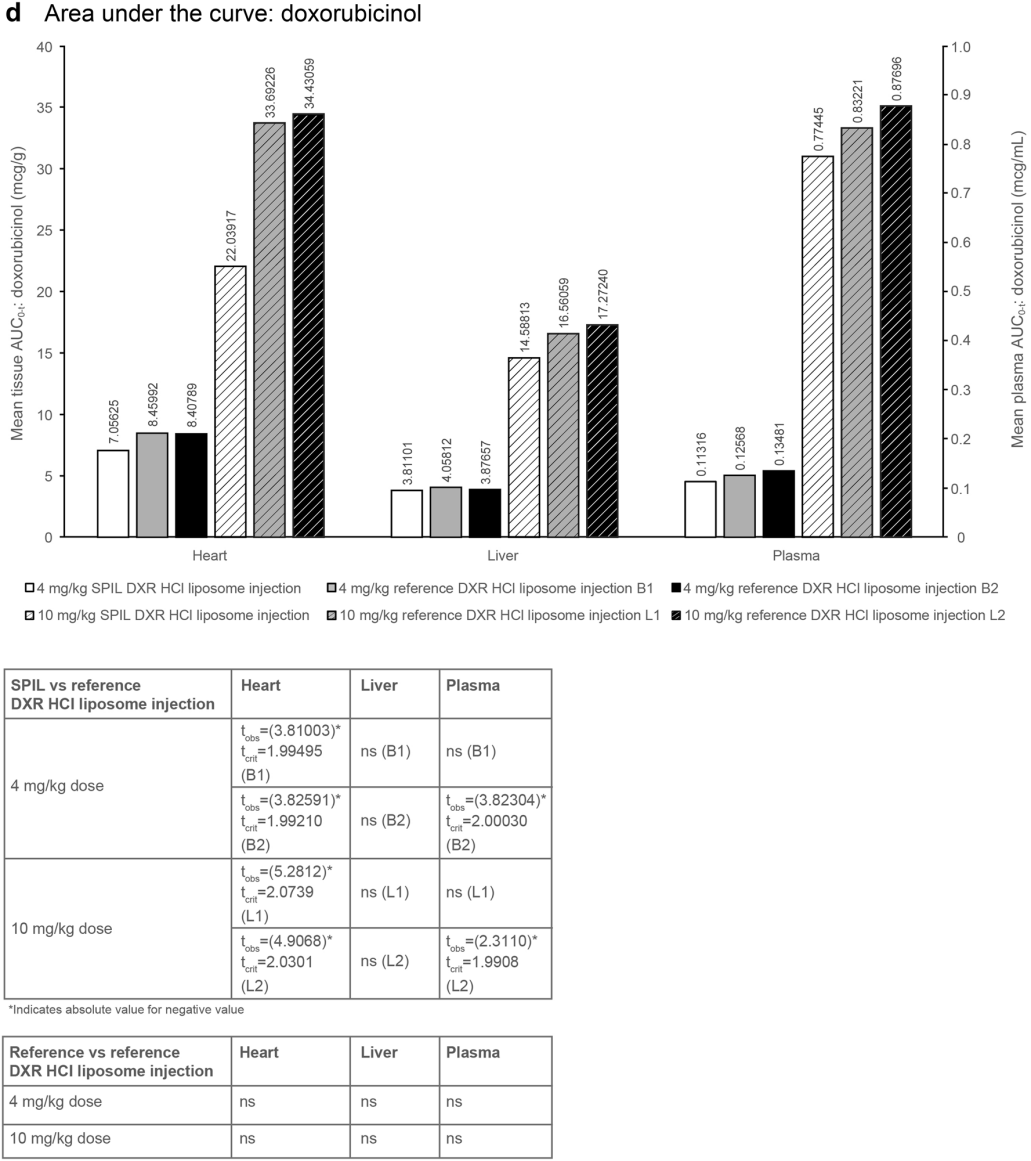
Plasma and tissue distribution of total DXR and doxorubicinol in Sprague–Dawley rats. **a** *C*_max_ and **b** AUC_0–*t*_ of total DXR after dosing with the SPIL or reference DXR HCl liposome injection. **c** *C*_max_ and **d** AUC_0–*t*_ of doxorubicinol after dosing with the SPIL or reference DXR HCl liposome injection. Ten animals were analysed per time point. Differences in *C*_max_ were analysed by unpaired *t*-test. *P* values ≤0.05 were considered significant. Differences in AUC_0–*t*_ were analysed by the Bailer–Satterthwaite method, and a difference was considered to be statistically significant when abs(*t*_obs_) ≥ *t*_crit_. *AUC* area under the concentration–time curve, *C*_*max*_ mean peak concentration, *B1* batch 1, *B2* batch 2, *DXR* doxorubicin, *L1* lot 1, *L2* lot 2, *ns* not significant, *SPIL* Sun Pharmaceutical Industries Limited

Mean plasma *C*_max_ and AUC_0–*t*_ values for Sprague–Dawley rats, following a single intravenous injection with either the SPIL or reference DXR HCl liposome injection, are shown in Fig. 2. At 4 mg/kg, the plasma distribution of the SPIL DXR HCl liposome injection was compared with 2 different batches of the reference product [batch 1 (B1) and batch 2 (B2)] and, at 10 mg/kg, with 2 different lots of the reference product [lot 1 (L1) and lot 2 (L2)]. The different batches and lots of the reference product were also compared with each other.

#### Maximum concentration

In plasma, the mean *C*_max_ values of total DXR were comparable between all three groups [SPIL and both batches (B1 and B2) of the reference product] at 4 mg/kg. The mean plasma *C*_max_ of encapsulated DXR was significantly lower for the SPIL DXR HCl liposome injection compared with B1 and B2 of the reference product (*p* = 0.0126 and *p* = 0.0001); however, the *C*_max_ values of B1 and B2 were also significantly different when compared with each other (*p* = 0.0240). The mean plasma *C*_max_ of free DXR was significantly higher for the SPIL DXR HCl liposome injection than for reference DXR HCl liposome injection B2 at 4 mg/kg (*p* = 0.0354). Also at 4 mg/kg, the mean plasma *C*_max_ of doxorubicinol was significantly lower for the SPIL DXR HCl liposome injection compared with reference DXR HCl liposome injection B2 (*p* = 0.0217; Fig. 2c). The mean plasma *C*_max_ values of all four analytes (total, free and encapsulated DXR, and doxorubicinol) were comparable between the SPIL and both lots (L1 and L2) of the reference DXR HCl liposome injection, at 10 mg/kg (Fig. 2a).

#### Area under the curve

The mean plasma AUC_0–*t*_ values of total DXR were comparable between the SPIL and reference DXR HCl liposome injection B1 and B2 at 4 mg/kg. However, the AUC_0–*t*_ values of total DXR for the SPIL DXR HCl liposome injection were significantly lower compared with reference DXR HCl liposome injection L1 at 10 mg/kg (*t*_crit_ = 1.9955 and *t*_obs_ = −2.7108; Fig. 2b). The mean plasma AUC_0–*t*_ values for doxorubicinol for the SPIL DXR HCl liposome injection were significantly lower compared with reference product B2 at 4 mg/kg (*t*_crit_ = 2.00030 and *t*_obs_ = −3.82304) and reference product L2 at 10 mg/kg (*t*_crit_ = 1.9908 and *t*_obs_ = −2.3110). However, this was not the case for the SPIL DXR HCl liposome injection compared with reference product B1 at 4 mg/kg and reference product L1 at 10 mg/kg, where plasma AUC_0–*t*_ values were comparable (Fig. 2d). Mean AUC_0–*t*_ for free DXR was significantly higher for SPIL compared with reference DXR HCl liposome injection B2 at 4 mg/kg (*t*_crit_ = 2.0181 and *t*_obs_ = 2.0467). However, mean AUC_0–*t*_ for encapsulated DXR was significantly lower for SPIL compared with either reference DXR HCl liposome injection B1 or B2, at 4 mg/kg. These findings suggest that at this dose, there may be a difference between the SPIL and reference DXR HCl liposome injections in plasma distribution of encapsulated versus free forms of DXR.

### Tissue distribution of DXR in Sprague–Dawley rats

#### Maximum concentration: total DXR

Overall, the *C*_max_ values of total DXR for most tissues were comparable between SPIL and each batch and lot of the reference DXR HCl liposome injection (Fig. 2a). The mean *C*_max_ values of total DXR were significantly lower in the heart for the SPIL DXR HCl liposome injection compared with reference product B2, at 4 mg/kg, and reference product L2, at 10 mg/kg (*p* = 0.0351 and *p* = 0.0137, respectively). The mean *C*_max_ values of total DXR in the kidney were significantly lower for the SPIL DXR HCl liposome injection compared with reference product B2, at 4 mg/kg, (*p* = 0.0089), and with reference product L1 and L2, at 10 mg/kg (*p* = 0.0044 and *p* = 0.0005). In lung tissue, mean *C*_max_ values of total DXR were also significantly lower for the SPIL than for the reference DXR HCl liposome injection L1 at 10 mg/kg (*p* = 0.0019); however, the *C*_max_ values of L1 and L2 were also significantly different in the lung (*p* = 0.0002). The mean *C*_max_ value of total DXR in the spleen was significantly higher for SPIL compared with one of the reference DXR HCl liposome injection lots (L1; *p* = 0.0155).

#### Area under the curve: total DXR

Overall exposure to total DXR in the heart and kidney was similarly lower for SPIL compared with either of the reference DXR HCl liposome injections at 4 and 10 mg/kg (Fig. 2b). This was also true in the liver at 4 mg/kg, with significantly lower AUC_0–*t*_ for SPIL compared with reference DXR HCl liposome injection B1 and B2; however, at 10 mg/kg, the SPIL DXR HCl liposome injection was comparable with reference product L1 and L2, in the liver. There was some evidence of differences between the AUC_0–*t*_ values for SPIL compared with those for one of the batches and lots of the DXR HCl liposome injection in some of the other tissues, but there was no obvious trend to this, and for some tissues, there was a significant difference between mean AUC_0–*t*_ values of reference product B1 and B2, and between mean AUC_0–*t*_ values of reference product L1 and L2.

#### Maximum concentration: doxorubicinol

The mean *C*_max_ of doxorubicinol was comparable between the SPIL and reference DXR HCl liposome injections in liver tissue, after 4 mg/kg. However, the mean liver *C*_max_ was significantly lower for 10 mg/kg SPIL compared with reference DXR HCl liposome injection L1 and L2 (*p* = 0.0274 and *p* = 0.0315). The mean heart *C*_max_ of doxorubicinol was significantly lower for SPIL compared with reference product B1 and B2 at 4 mg/kg, and compared with reference product L1 and L2 at 10 mg/kg (Fig. 1c).

#### Area under the curve: doxorubicinol

The mean liver AUC_0–*t*_ values of doxorubicinol were comparable for the SPIL and reference DXR HCl liposome injections after either 4 or 10 mg/kg (Fig. 2d). A significantly lower heart AUC_0–*t*_ value was observed for SPIL compared with reference product B1 and B2 at 4 mg/kg, and compared with reference product L1 and L2 at 10 mg/kg.

### Ratios of free to encapsulated DXR

The ratios of estimated free to encapsulated DXR for syngeneic fibrosarcoma-bearing mice and Sprague–Dawley rats, following a single intravenous injection with either the SPIL or reference DXR HCl liposome injection, are shown in Table 2. In syngeneic fibrosarcoma-bearing mice, the ratios of estimated free to encapsulated DXR for all the tissues were comparable between the two products at 2.4 mg/kg (Table 2a). At 6 mg/kg, the ratios of estimated free to encapsulated DXR were also comparable for all the tissues between the two products, except for minor differences in bone marrow, tumour and skin (Table 2b).

**Table 2:** Ratios of free to encapsulated DXR

Tissues	Free drug/encapsulated drug *C*_max_			Free drug/encapsulated drug AUC_0–*t*_		
	SPIL DXR HCl liposome injection	Reference DXR HCl liposome injection		SPIL DXR HCl liposome injection	Reference DXR HCl liposome injection	
(a) Ratios of free to encapsulated DXR in syngeneic fibrosarcoma-bearing mice (2.4 mg/kg dose)						
Plasma	0.03	0.03		0.04	0.03	
Bone marrow	1.4	1.5		5.3	4.4	
Tumour	0.1	0.1		0.2	0.4	
Skin	0.7	1.2		1.3	1.2	
Kidney	0.04	0.04		0.1	0.1	
Heart	0.2	0.1		0.1	0.1	
Spleen	0.08	0.1		0.1	0.1	
Lung	0.1	0.08		0.1	0.4	
Liver	0.08	0.06		0.2	0.1	
(b) Ratios of free to encapsulated DXR in syngeneic fibrosarcoma-bearing mice (6 mg/kg dose)						
Plasma	0.02	0.02		0.03	0.03	
Bone marrow	1.4	0.5		3.9	1.9	
Tumour	0.3	0.1		0.4	0.2	
Skin	2.2	0.8		1.8	0.9	
Kidney	0.09	0.09		0.1	0.09	
Heart	0.3	0.3		0.5	0.4	
Spleen	0.5	0.4		0.8	0.8	
Lung	0.2	0.1		0.2	0.1	
Liver	0.2	0.2		0.5	0.4	

Tissues	Free drug/encapsulated drug *C*_max_			Free drug/encapsulated drug AUC_0–*t*_		
	SPIL DXR HCl liposome injection	Reference DXR HCl liposome injection (batch 1)	Reference DXR HCl liposome injection (batch 2)	SPIL DXR HCl liposome injection	Reference DXR HCl liposome injection (batch 1)	Reference DXR HCl liposome injection (batch 2)
(c) Ratios of free to encapsulated DXR in Sprague–Dawley rats (4 mg/kg dose)						
Plasma	0.02	0.01	0.01	0.02	0.02	0.02
Bone marrow	1.4	1.6	2.3	1.6	2.2	1.8
Skin	1.2	0.8	1.5	3.3	2.0	2.0
Kidney	0.7	0.4	0.3	1.4	1.0	0.7
Heart	0.5	0.4	0.4	1.1	1.2	1.1
Spleen	0.7	0.3	0.3	1.3	0.9	0.4
Lung	0.3	0.2	0.3	0.2	0.1	0.1
Liver	0.3	0.2	0.2	0.8	0.5	0.5

Tissues	Free drug/encapsulated drug *C*_max_			Free drug/encapsulated drug AUC_0–*t*_		
	SPIL DXR HCl liposome injection	Reference DXR HCl liposome injection (lot 1)	Reference DXR HCl liposome injection (lot 2)	SPIL DXR HCl liposome injection	Reference DXR HCl liposome injection (lot 1)	Reference DXR HCl liposome injection (lot 2)
(d) Ratios of free to encapsulated DXR in Sprague–Dawley rats (10 mg/kg dose)						
Plasma	0.01	0.01	0.01	0.02	0.02	0.02
Bone marrow	2.9	2.7	5.0	3.5	4.4	5.9
Skin	9.9	8.4	11.1	14.1	10.1	8.0
Kidney	0.4	0.2	0.2	1.5	0.8	0.7
Heart	0.7	0.6	0.6	1.3	1.2	1.2
Spleen	0.7	0.6	0.4	2.4	1.9	1.0
Lung	0.3	0.3	0.2	0.3	0.3	0.3
Liver	0.3	0.3	0.3	0.6	0.5	0.4

*AUC* area under the concentration–time curve, *C*_*max*_ mean peak concentration, *DXR* doxorubicin, *HCl* hydrochloride, *SPIL* Sun Pharmaceutical Industries Limited

In Sprague–Dawley rats, the ratios of estimated free DXR to encapsulated DXR in plasma and all tissues examined were generally comparable between the SPIL DXR HCl liposome injection and each of the two batches of the reference product at 4 mg/kg (Table 2c), and between the SPIL DXR HCl liposome injection and each of the two lots of the reference product at 10 mg/kg (Table 2d).

## Discussion

The availability of a generic DXR HCl liposome injection could potentially improve access to an important anticancer agent. Because the improved benefit-risk profile of Caelyx^®^ results from its altered tissue distribution, a generic liposomal formulation of DXR must demonstrate a comparable plasma and tissue distribution [7]. As it is not possible to study tissue distribution in humans, in vivo nonclinical data are crucial to the regulatory decision of whether to approve a generic DXR liposomal product [7].

The objectives of the studies presented in this paper were to compare the plasma and tissue distribution of the SPIL DXR HCl liposome injection with the reference DXR HCl liposome injection following single intravenous injection in murine models. To compare the two products in a tumour background, a syngeneic fibrosarcoma mouse model was used.

In syngeneic fibrosarcoma-bearing BALB/c mice, the ratios of estimated free to encapsulated DXR for all the tissues were comparable between the two products, except for minor differences in bone marrow, tumour, and skin at 6 mg/kg. This indicates that the SPIL and reference formulations have similar extents of absorption into the tissues and similar rates of drug release from liposomes. Despite some evidence of biological variations (especially in the liver and kidney), at 2.4 mg/kg, the SPIL and reference products showed generally comparable plasma and tissue distribution profiles in syngeneic fibrosarcoma-bearing BALB/c mice.

In Sprague–Dawley rats, the ratios of estimated free to encapsulated DXR for plasma and all tissues are comparable between the SPIL DXR HCl liposome injection and the two batches and lots of the reference product, indicating that the test and reference formulations have similar extents of absorption into the tissues and similar rates of drug release from liposomes. The minor differences observed between the SPIL and reference products in some tissues may be attributed to inherent biological variations of the test system, since two different batches of the reference DXR HCl liposome injection also showed some differences. The SPIL and reference products exhibited generally comparable plasma and tissue distribution profiles in Sprague–Dawley rats. Even two different batches and lots of the reference DXR HCl liposome injection failed to show comparable distribution of all the analytes in all the tissues, suggesting any variations of tissue distribution may be caused by the inherent biological fluctuation of the test system.

Based on *C*_max_ and mean AUC_0–*t*_ values, there was evidence that the SPIL DXR HCl liposome injection may lead to lower exposure of the cardiotoxic metabolite doxorubicinol in the heart in rats, and also some suggestion of the same in plasma and the liver, compared with the reference DXR HCl liposome injection. Doxorubicinol is thought to be responsible for the cardiotoxicity associated with DXR. The SPIL DXR HCl liposome injection may therefore be less cardiotoxic in rats. However, we acknowledge that this observation may not carry over to humans, and may be the result of inherent biological variation within the test system. There was also some evidence that the SPIL DXR HCl liposome injection leads to lower exposure of the cardiotoxic metabolite doxorubicinol in the liver (where DXR is metabolised) and potentially in plasma of mice, compared with the reference DXR HCl liposome injection. Because DXR is metabolised in the liver, lower levels of doxorubicinol in this tissue suggests that doxorubicinol should be lower in other tissues as well. This was not the case, so the doxorubicinol differences may be the result of inherent biological variation within the test system.

In conclusion, the plasma and tissue distribution profiles of the SPIL and reference DXR HCl liposome injections were shown to be generally comparable. Further studies, comparing the toxicology of SPIL DXR HCl liposome injection with Caelyx^®^ in murine models, will be published in the future.
